# Supplementary material for: Ten3–Lphn2-mediated target selection across the extended hippocampal network demonstrates a repeated strategy for circuit assembly
Source: Curr Biol. Author manuscript; Available in PMC 2026 Jun 10. (PMC13250448; doi:10.1016/j.cub.2026.04.038)
Supplement: 1 [file NIHMS2174506-supplement-1.pdf]

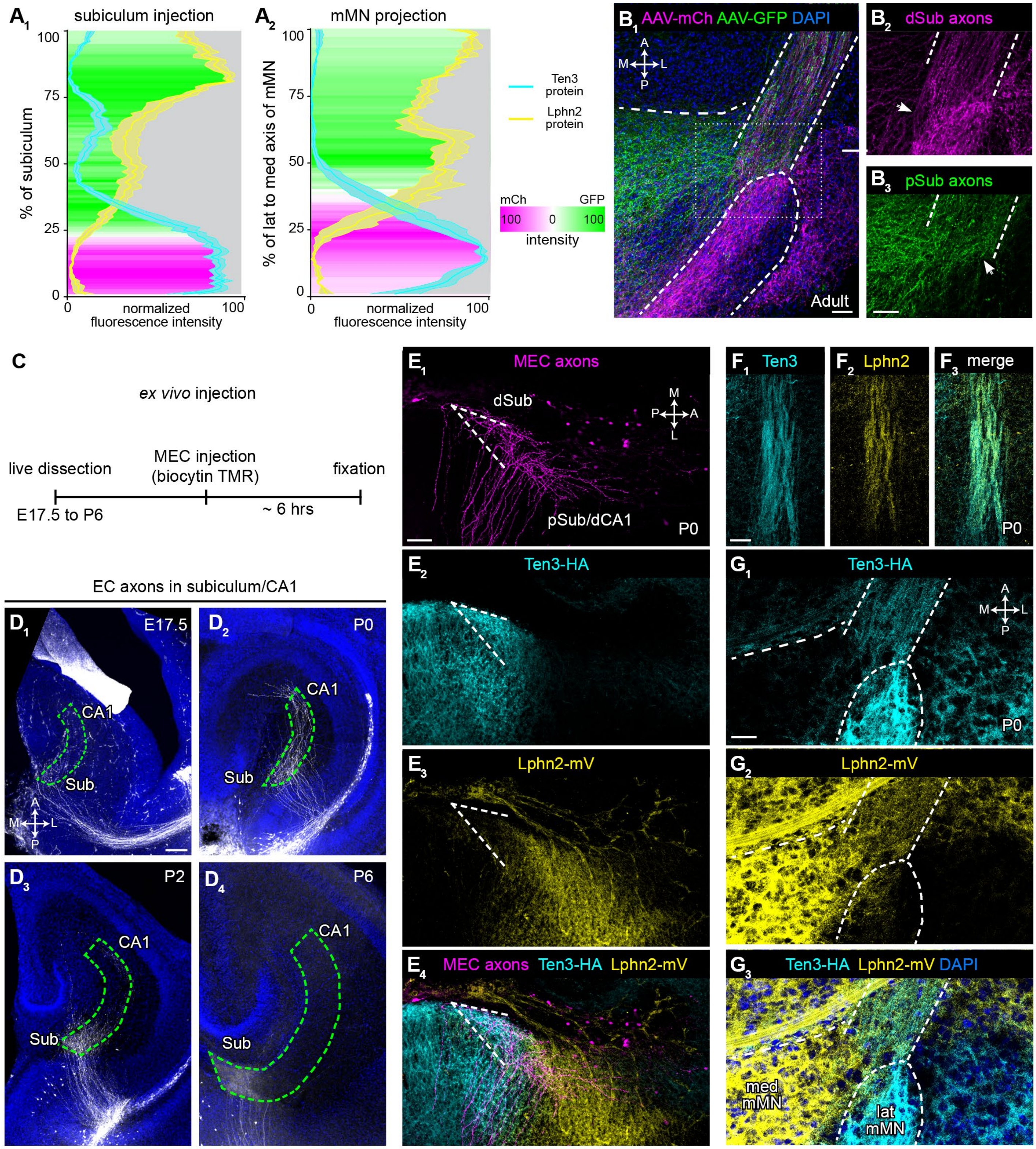

Figure S1. Additional data on axon tracing in adult and during development. Related to Figure 1.

(A) Quantitative depiction of dual-color anterograde tracing and Ten3/Lphn2 expression in subiculum→mMN projection in Figure 1B, C. (A_1) Normalized fluorescence intensity traces of Ten3 (cyan) and Lphn2 (yellow) protein expression along the distal-to-proximal axis of subiculum in P8 *$Ten3^{HA/HA}$;$Lphn2^{mVenus/mVenus}$* mouse (Figure $1B_1$) overlaid with dSub (mCherry, magenta) and pSub (GFP, green) injection site density heatmap (Figure $1C_1$). (A_2) a diagonal lat-mMN-to-med-mMN axis of mMN expression (Figure $1B_2$) overlaid with density heatmap of projections from dSub (magenta) and pSub (GFP; Figure $1C_2$). The correspondence between axon intensity at the injection and projection sites and Ten3/Lphn2 expression supports the 'Ten3→Ten3, Lphn2→Lphn2' connectivity rule.

(B) Additional representative image of topographic projections in lat-mMN and med-mMN (B_1; from dual dSub and pSub injections), show axons do not segregate until reaching the target and then turn (arrows) to fill $Ten3^+$ (B_2) and $Lphn2^+$

subfields (B_3). B_2 and B_3 are from dotted rectangle in B_1, labeled only with mCherry or GFP channels, respectively. Scale bar, 50 μm.
(C) Schematic for *ex vivo* injections for developmental tracing of entorhinal cortex→subiculum projection.
(D) Representative images of projection of entorhinal cortex axons into the molecular layer of subiculum in wildtype animals at E17.5 (D_1), P0 (D_2), P2 (D_3), and P6 (D_4). Axons begin to invade subiculum at E17.5 and progressively increase in density through P6. Scale bar, 100 μm.
(E) Representative images of MEC axons (magenta) at P0 entering Ten3-high dSub (cyan) and turning before proceeding to Lphn2-high pSub (yellow) at P0. The three images are from the same confocal section showing axons (E_1), Ten3 protein (E_2), Lphn2 protein (E_3), and merge (E_4) channels. Scale bar, 50 μm.
(F) Representative images of subiculum axon tract at P0 along the path to mMN with extensive intermingling of $Ten3^+$ (cyan) and $Lphn2^+$ (yellow) axons in a $Ten3^{HA/HA};Lphn2^{mVenus/mVenus}$ mouse. The three images are from the same confocal section showing Ten3 (F_1), Lphn2 (F_2), and merge (F_3) channels. Scale bar, 50 μm.
(G) Representative images of subiculum axon tract at P0, entering mMN target where axons experience $Ten3^+$ and $Lphn2^+$ subfields simultaneously. Ten3 (cyan) and Lphn2 (yellow) axons remain intermingled in the tract up until terminal projections in the target. The three images are from the same confocal section showing Ten3 (G_1), Lphn2 (G_2), and merge (G_3) channels. Scale bar, 25 μm.

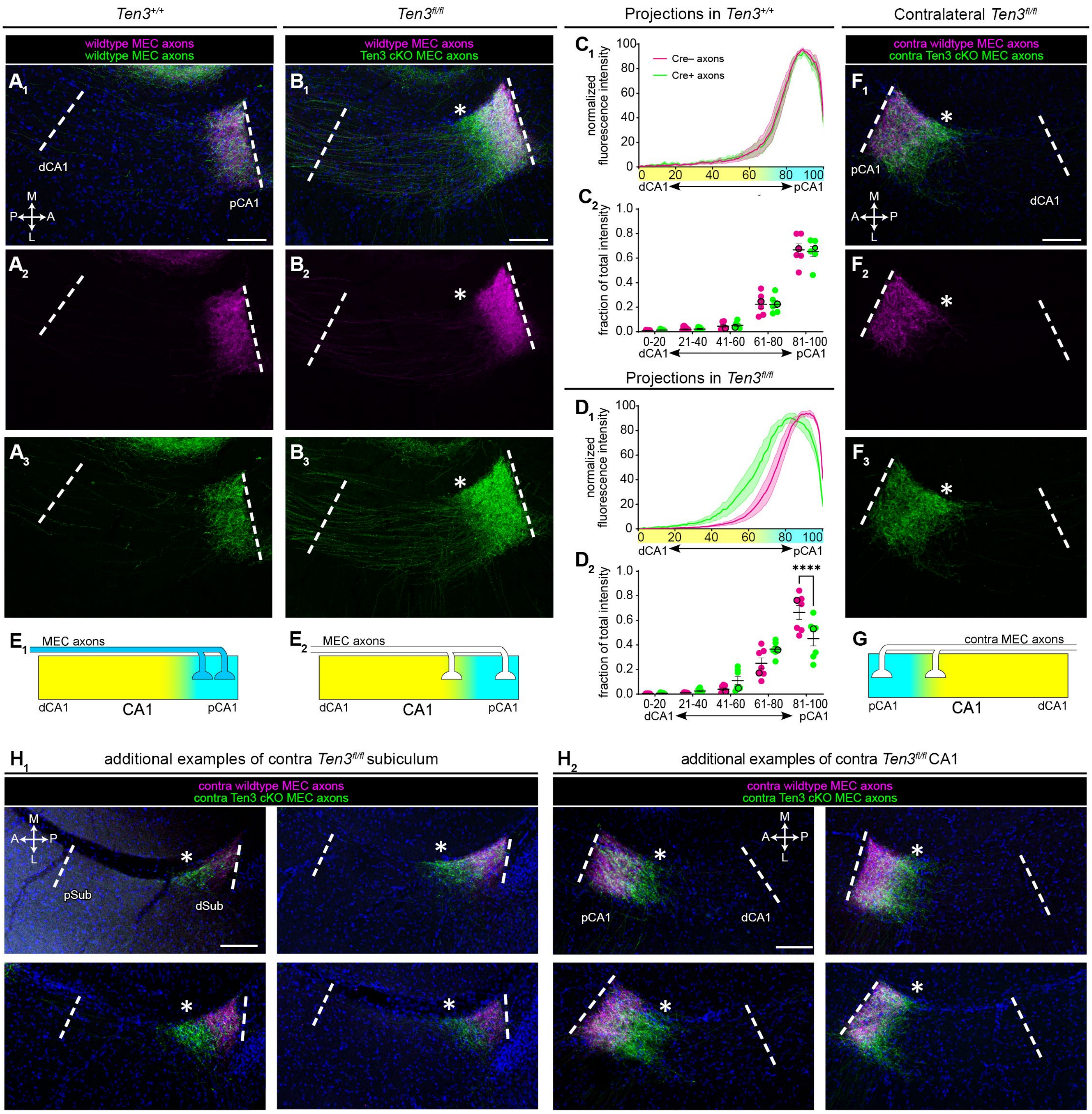

Figure S2. Additional analyses of the MEC axon projection. Related to Figure 2.

(A) Representative images of projection of Cre⁻ axons (magenta), and Cre⁺ axons (green) into molecular layer of CA1 in *Ten3*$^{+/+}$ controls. The three images are from the same confocal section showing merge (A_1), magenta only (A_2), or green only (A_3) channels. Corresponding injection is in Figure 2C.

(B) Same as A but for *Ten3*$^{fl/fl}$ mice. *Ten3* cKO axons in *Ten3*$^{fl/fl}$ mice spread beyond the Cre⁻ control axons towards dCA1 (asterisk, in identical position across three images). Corresponding injection is in Figure 2E.

(C_1) Normalized fluorescence intensity traces of the Cre⁻ (magenta) and Cre⁺ (green) projections along the distal→proximal axis of CA1 in *Ten3*$^{+/+}$ controls (n = 6 mice). Injection positions are in Figure 2B. Ten3 (cyan) and Lphn2 (yellow) expression is represented along the x-axis. Mean (dark line) ± SEM (shaded area).

(C_2) Fraction of total projection intensity (same as C_1) in 20% bins of CA1 axis of the Cre⁻ (magenta) and Cre⁺ (green) projections along the distal–proximal axis of CA1 in *Ten3*$^{+/+}$ controls (n = 6 mice). Black outlines indicate representative animal in A. Mean ± SEM. Two-way ANOVA corrected for multiple comparisons (Šídák correction), no significant differences.

(D) Same as C but for *Ten3*$^{fl/fl}$ (n = 7) mice. **** $p < 0.0001$.

(E) Schematic summary of target selection of MEC axons in CA1 in control (E_1) and *Ten3*^{MEC}-cKO (E_2) based on results in A–D.
(F) Same as B, but for projections into contralateral CA1. Contralateral Cre$^+$, *Ten3*^{MEC}-cKO axons spread beyond the contralateral Cre$^-$ control axons towards dCA1 (asterisk, in identical position across three images). Corresponding injection is in Figure 2K.
(G) Schematic summary of target selection of MEC axons in contralateral CA1 in *Ten3*^{MEC}-cKO mice.
(H) Four additional examples of contralateral projections in subiculum (H_1) and CA1 (H_2) of *Ten3*^{MEC}-cKO. Asterisks indicate GFP$^+$ axon mistargeting.
Scale bars, 100 μm.

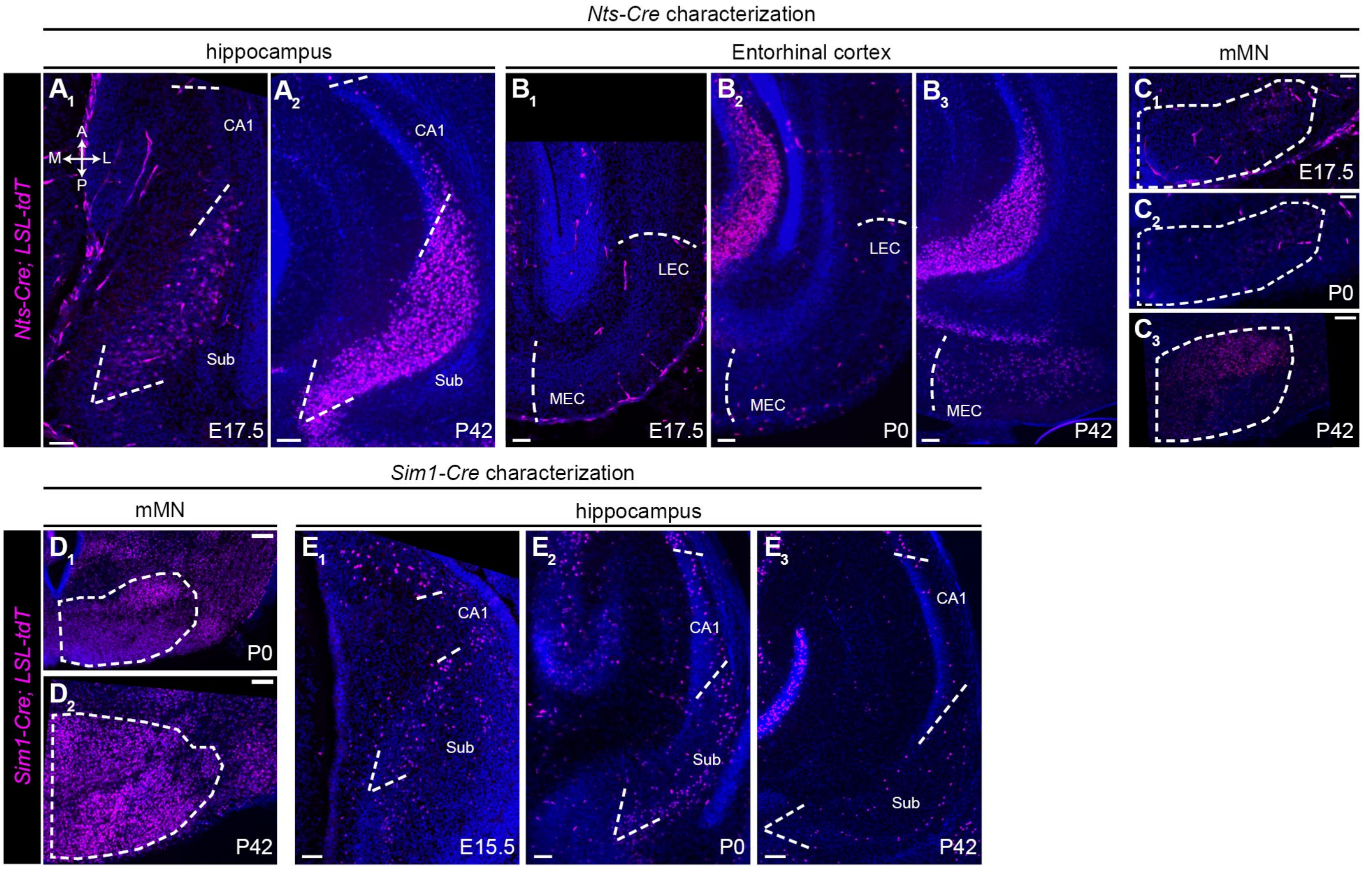

Figure S3. *Nts-Cre* and *Sim1-Cre* characterizations. Related to Figures 3–7.

(A) Expression of *Nts-Cre* in subiculum and CA1, assessed by a nuclear tdTomato Cre reporter at E17.5 (A_1) and P42 (A_2). P0 data is in Figure 3A. The Cre reporter is sparsely expressed in subiculum at E17.5 and is denser by P0. Little CA1 expression is found even in adult. Scale bar, 50 µm (A_1), 100 µm (A_2).

(B) Same as A, except in entorhinal cortex with the addition of P0 (B_2). Expression is sparse in entorhinal cortex during development, ensuring target-only deletions in our examination of the MEC→dSub projection (Figure 3). Scale bar, 50 µm (B_1, B_2), 100 µm (B_3).

(C) Same as A, except in medial mammillary nucleus (mMN) with the addition of P0 (C_2). Expression is sparse in mMN during development, ensuring axon-only deletions in our examination of the subiculum→mMN projections (Figures 4 and 6). Scale bar, 50 µm (C_1, C_2), 100 µm (C_3).

(D) Expression of *Sim1-Cre* in mMN, assessed by a nuclear tdTomato Cre reporter at P0 (D_1) and P42 (D_2). E15.5 data is in Figure 5A. The Cre reporter is densely expressed in mMN by E15.5. Scale bars, 50 µm (D_1), 100 µm (D_2).

(E) Same as D, except in subiculum with the addition of E15.5 (E_1). Expression is sparse in subiculum during development and even in adults, ensuring target-only deletions in our examination of the subiculum→mMN projections (Figures 5 and 7). Scale bar, 50 µm (E_1, E_2), 100 µm (E_3).

All panels show endogenous tdTomato fluorescence, except for E17.5 *Nts-Cre* panels which are stained with RFP antibody (A_1, B_1, C_1).

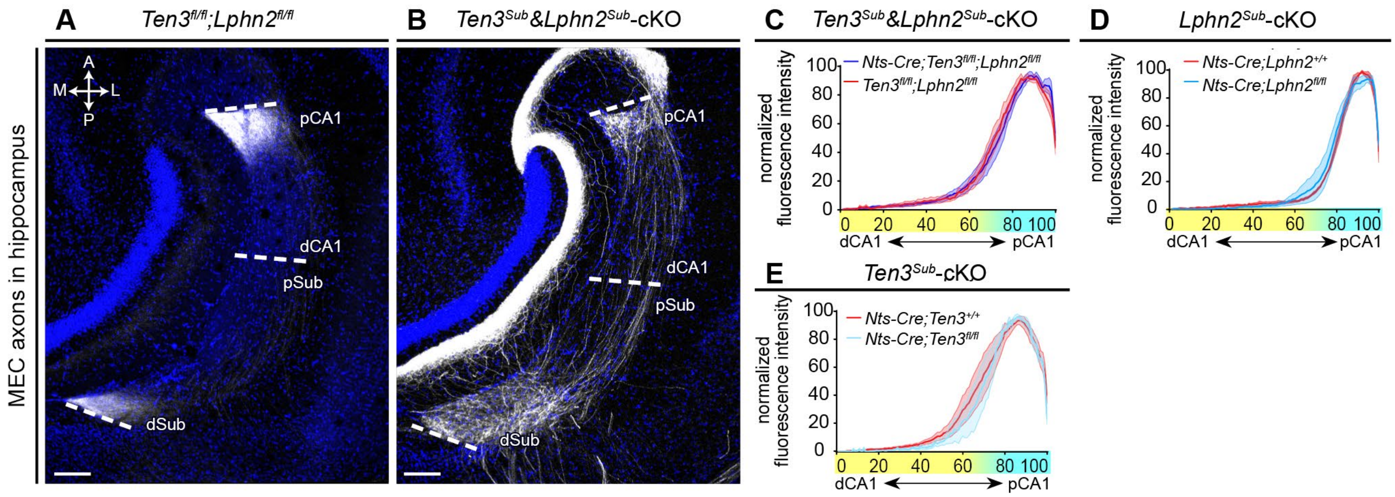

Figure S4. Normal targeting of MEC→pCA1 when *Ten3&Lphn2* were conditionally knocked out in subiculum. Related to Figure 3.

(A) Representative image of MEC projections into molecular layer of CA1 and subiculum for controls. This is a larger field of view of Figure 3E to show that the same MEC axon population target both subiculum and CA1 with their terminals restricted to dSub and pCA1. Corresponding injection is in Figure 3D. Scale bar, 100 μm.

(B) Same as A, but for *Ten3^{Sub}&Lphn2^{Sub}*-cKO mice. This is a larger field of view of Figure 3G$_1$. MEC axons spread towards pSub in subiculum but are restricted to pCA1 in CA1 as in control animals. Corresponding injection is in Figure 3F.

(C) Normalized fluorescence intensity traces of MEC axon projections along the distal-to-proximal axis of CA1 internal controls (n = 6; red) and *Ten3^{Sub}&Lphn2^{Sub}*-cKO mice (n = 5; blue). Injection positions are in Figure 3C. Ten3 (cyan) and Lphn2 (yellow) expression is represented along the x-axis. Mean (dark line) ± SEM (shaded area).

(D) Same as C, but for control (n = 3; red) and *Lphn2^{Sub}*-cKO (n = 5; blue) mice.

(E) Same as C, but for control (n = 5; red) and *Ten3^{Sub}*-cKO (n = 4; blue) mice.

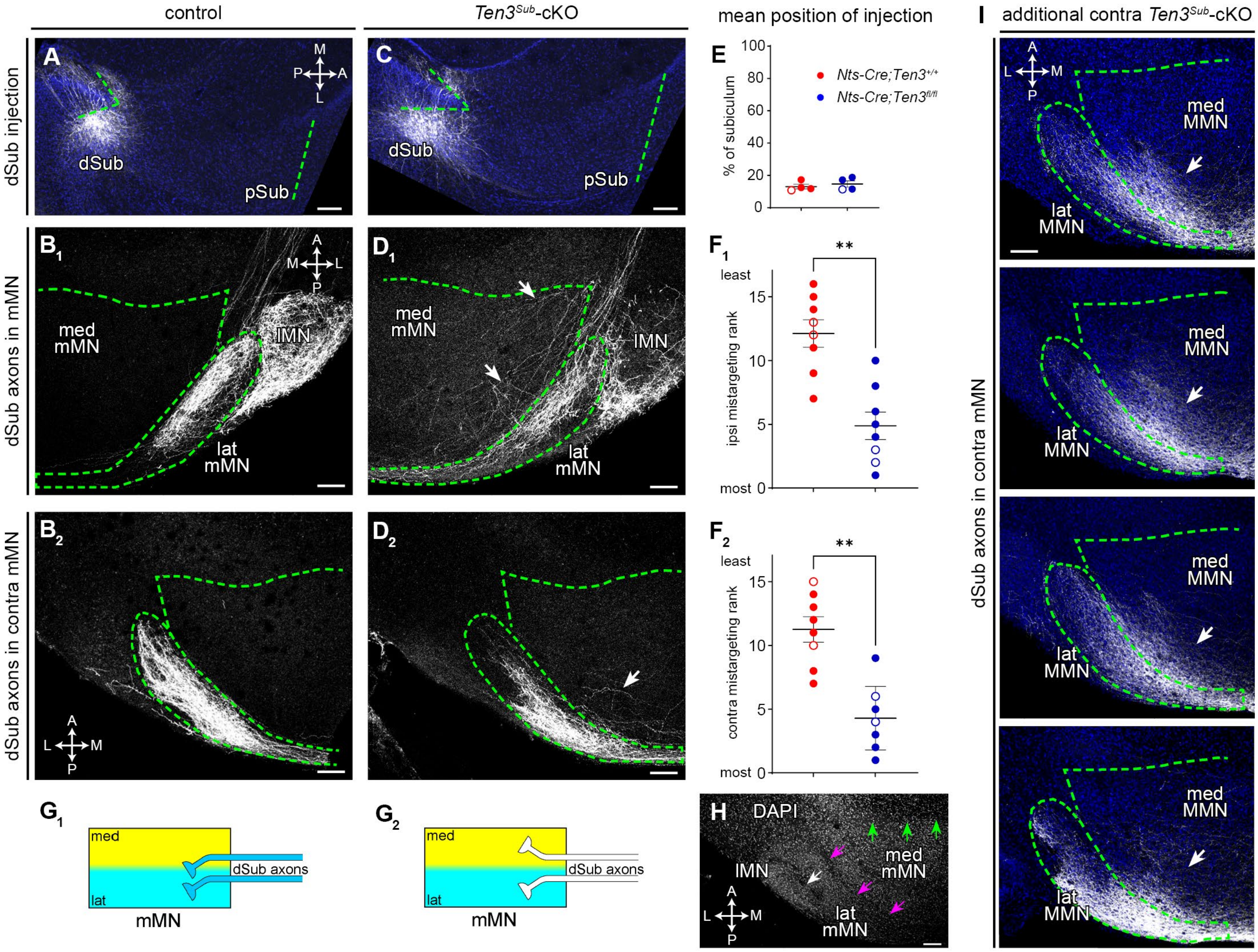

Figure S5. Additional *Ten3* axon deletion data to examine the dSub→lat-mMN projection. Related to Figure 4.
In an independent set of experiments, we examined the phenotypes of dSub→lat-mMN projection in *Ten3^{Sub}*-cKO mice. Here, mistargeting of dSub axons into med-mMN in control and *Ten3^{Sub}*-cKO mice were rank-ordered by an experimenter blind to the genotype, based on the relative intensity of dSub axons in med-mMN and lat-mMN (to account for variations in injection intensity of anterograde tracers).

(A) Representative image of the *AAV-CreON-mCh* (gray) injection site of controls show the virus is restricted to the most distal region of subiculum. Injection corresponds to animal in (B).

(B) Representative images of projection into ipsilateral (B_1) and contralateral (B_2) mMN of controls. Labeling in lateral mammillary nucleus (lMN) are unrelated axons from pre-subiculum.

(C, D) Same as A, B, but for *Ten3^{Sub}*-cKO mice. Even the most distal subiculum axons show aberrant branches in med-mMN (arrows in D).

(E) Mean positions of injection sites along the distal-to-proximal axis of subiculum show no differences between control (n = 4, red) and *Ten3^{Sub}*-cKO (n = 4, blue). Open circles indicate representative image in A, C. Mean ± SEM. Mann-Whitney test, no significant differences.

(F) Rank according to severity of mistargeting for control (n = 8 section, red) and *Ten3^{Sub}*-cKO (n = 8 sections, blue) in ipsilateral (F_1) and contralateral (F_2) mMN. Contralateral cKO has only n = 7 cKO sections. Open circles indicate representative animals in B, D. Ranked from most severe (1) to least severe (16). Mean ± SEM. Mann-Whitney test, ** $p < 0.01$.

(G) Schematic summary of target selection of dSub axons in contralateral mMN of control (G_1) and *Ten3^{Sub}*-cKO (G_2) mice based on results in A–F.

(H) Representative image of DAPI counterstain in mMN illustrates borders of sub-nuclei used to select regions of interest. Border of lMN (not studied) and lat-mMN (white arrow). Border of $Ten3^+$ lat-mMN and $Lphn2^+$ med-mMN (magenta arrows). Border of med-mMN and pre-mammillary nucleus (green arrows). Same section as Figure 4I.

(I) Four additional examples show mistargeting (arrows) of contralateral dSub→lat-mMN in *Ten3^{Sub}*-cKO. Corresponding injections in Figure 4B.

Scale bars, 100 μm.

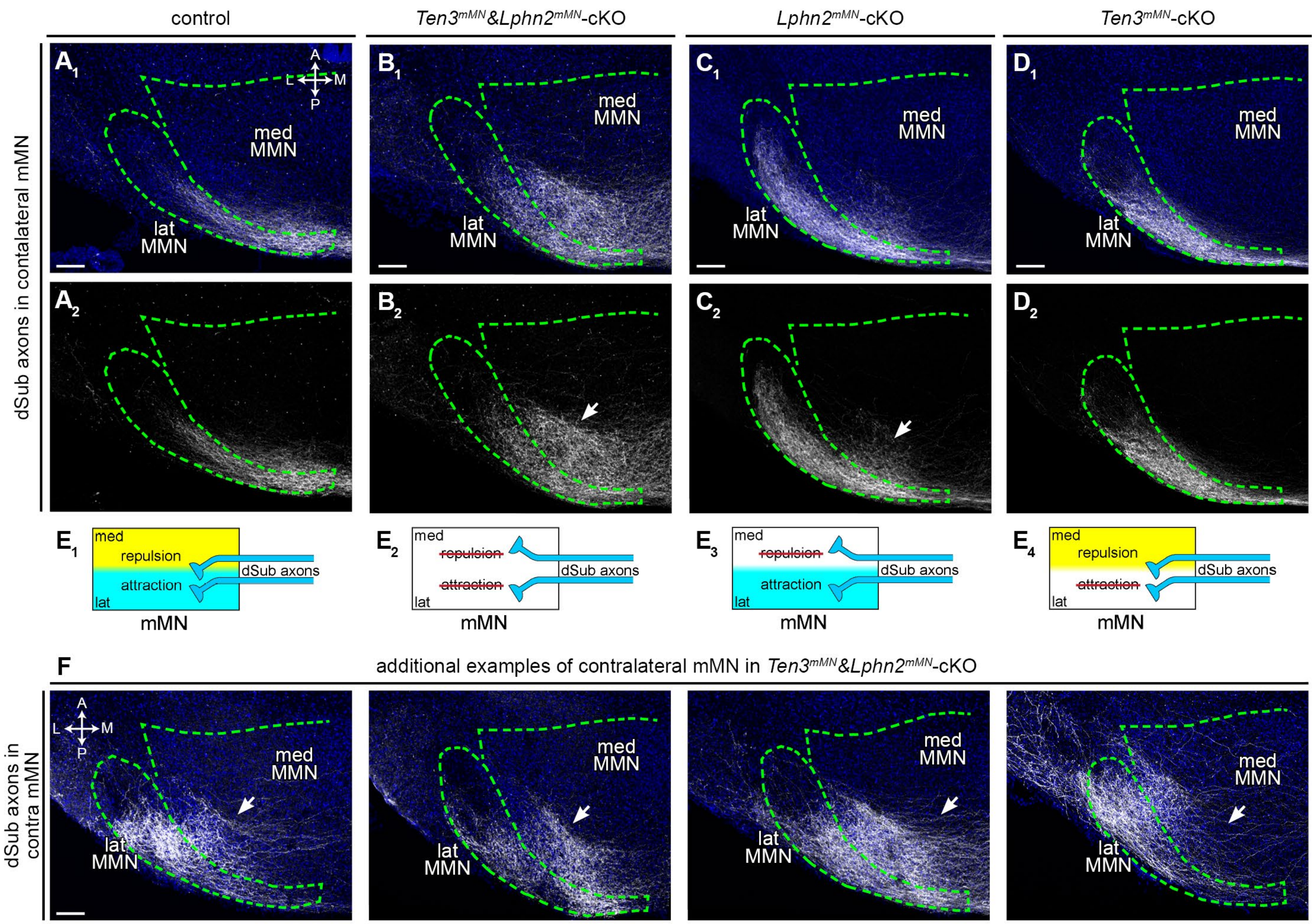

Figure S6. Analysis of contralateral dSub→lat-mMN projections in target deletion conditions. Related to Figure 5.
(A) Representative images of projection of dSub axons into contralateral medial mammillary nucleus of control mice (A_1). Bottom panel shows axons without DAPI counterstain (A_2). Same animal as Figure 5D, E.
(B–D) Same as A, but for genotypes indicated above. dSub axons in *Ten3*^{mMN}&*Lphn2*^{mMN}-cKO (B) and *Lphn2*^{mMN}-cKO (C) mice spread medially outside of contralateral lat-mMN (arrows in B_2, C_2). Same as animal of corresponding genotype in Figure 5F–K.
(E) Schematic summary of target selection of dSub axons in contralateral mMN in control (E_1) and when *Ten3* (E_4), *Lphn2* (E_3), or both (E_2) were conditionally knocked out in mMN.
(F) Four additional examples show mistargeting (arrows) of contralateral dSub→lat-mMN projections in *Ten3*^{mMN}&*Lphn2*^{mMN}-cKO. Corresponding injections in Figure 5C.
Scale bars, 100 μm.

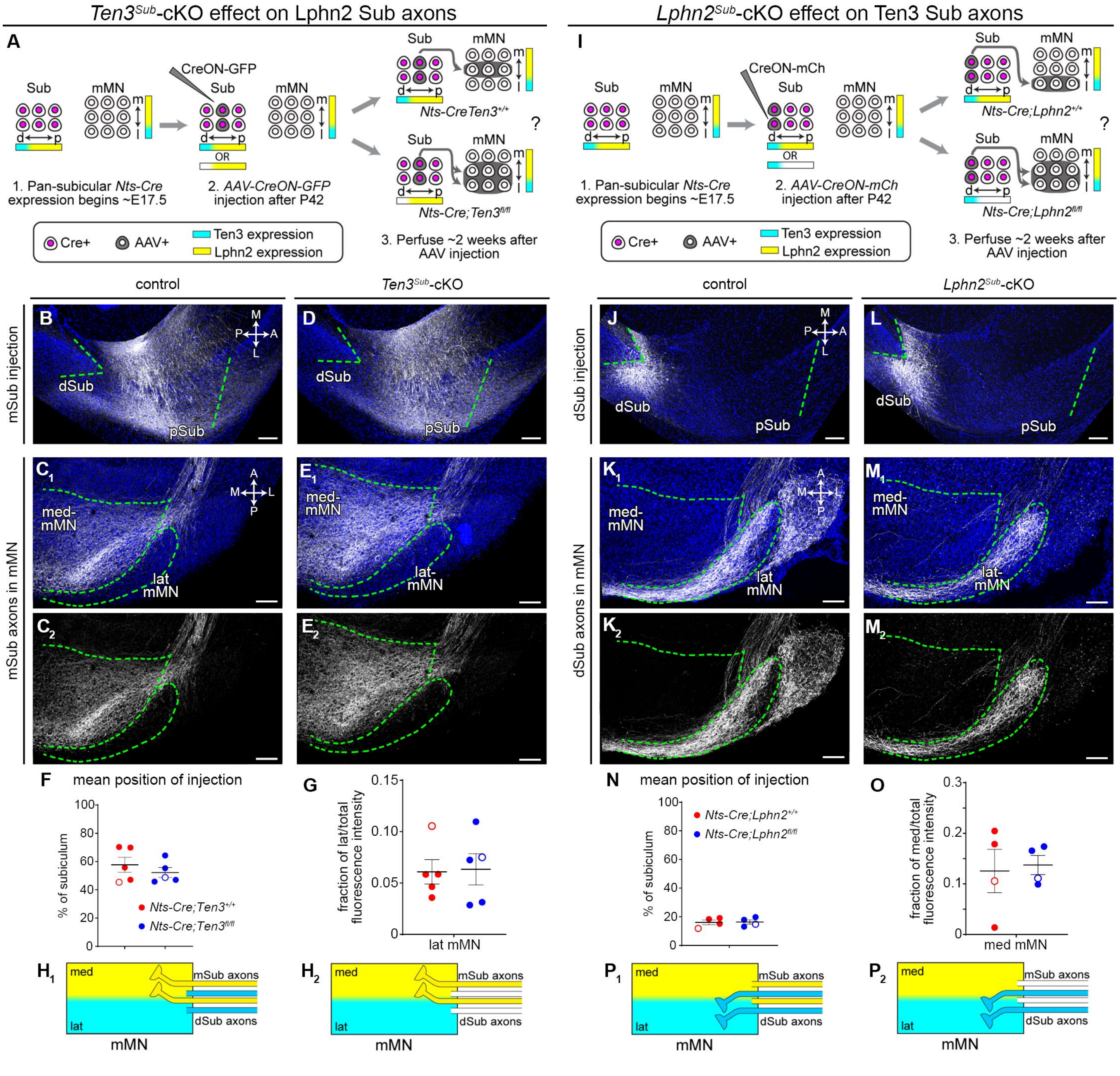

Figure S7. Lack of evidence for axon-axon interaction between Ten3-high dSub and Lphn2-high pSub axons in target selection of mMN. Related to Figures 4 and 6.

(A) Injection strategy and potential results for tracing functionally wildtype pSub (Lphn2-high) axons into medial mammillary nucleus (mMN) of *Nts-Cre;Ten3*$^{+/+}$ control (top right) and *Ten3*^{Sub}-cKO mice (bottom right).

(B) Representative image of the *AAV-CreON-GFP* (gray) injection site of controls show the virus is restricted to proximal region of subiculum. Injection corresponds to animal in (C).

(C) Representative images of projection of pSub axons (gray) into mMN of controls (C_1). Bottom panel shows axons without DAPI counterstain (C_2).

(D, E) Same as B, C, but for *Ten3*^{Sub}-cKO. Lphn2-high pSub axon targeting is unaffected by *Ten3* deletion in subiculum axons.

(F) Mean positions of injection sites along the proximal-distal axis of subiculum show no differences between controls (n = 5, red) and *Ten3*^{Sub}-cKO (n = 5, blue). Open circles indicate representative animals in B, D. Mean ± SEM. Mann-Whitney.

(G) Fraction of total projection intensity in lat-mMN of the pSub axons in controls (n = 5, red) and *Ten3*^{Sub}-cKO (n = 5, blue). Open circles indicate representative animals in C, E. Mean ± SEM. Mann-Whitney, no significant differences.

(H) Schematic summary of target selection of pSub axons in mMN of control (H_1) and *Ten3*^{Sub}-cKO (H_2) mice based on results in B–G. For clarity, we truncated the dSub axons in which *Ten3* was deleted.

(I–P) Same as A–H, but for functionally wildtype dSub (Ten3-high) *AAV-CreON-mCh* (gray) injections into *Nts-Cre;Lphn2*$^{+/+}$ control (n = 4, red) and *Lphn2*^{Sub}-cKO mice (n = 4, blue). Mean ± SEM. Mann-Whitney, no significant differences.
Scale bars, 100 μm.

				P0 Injection		Adult injection	
**Figure**	**Panel**	**Genotype**	**Designation**	**Region**	**Virus**	**Region**	**Virus/Tracer**
**Figure 1**	**B**	$Ten3^{HA/HA};Lphn2^{mVenus/mVenus}$	—	—	—	—	—
	**C**	wildtype	—	—	—	dSub<br>pSub	*AAV8-ChR2-mCherry*<br>*AAV8-ChR2-GFP*
	**D–E**	CD1 wildtype	—	—	—	—	—
**Figure 2**	**C–D**	CD1 wildtype	control ($Ten3^{+/+}$)	MEC	*LV-CAG-Cre-GFP*	MEC	*AAV8-CreON-GFP*<br>*AAV8-CreOFF-mCherry*
	**E–F, K–L**	$Ten3^{fl/fl}$	$Ten3^{MEC}$*-cKO*	MEC	*LV-CAG-Cre-GFP*	MEC	*AAV8-CreON-GFP*<br>*AAV8-CreOFF-mCherry*
**Figure 3**	**A**	*Nts-Cre;Ai75D/+*	*Nts-Cre;LSL-ntdT* [1]	—	—	—	—
	**D–E**	$Ten3^{fl/fl};Lphn2^{fl/fl}$	control	—	—	MEC	*AAV8-CreOFF-mCherry*
	**F–G**	$Nts\text{-}Cre;Ten3^{fl/fl};Lphn2^{fl/fl}$	$Ten3^{Sub}$&$Lphn2^{Sub}$-cKO	—	—	MEC	*AAV8-CreOFF-mCherry*
	**H–I**	$Nts\text{-}Cre;Ten3^{+/+};Lphn2^{fl/fl}$	$Lphn2^{Sub}$-cKO	—	—	MEC	*AAV8-CreOFF-mCherry*
	**J–K**	$Nts\text{-}Cre;Ten3^{fl/fl};Lphn2^{+/+}$	$Ten3^{Sub}$-cKO	—	—	MEC	*AAV8-CreOFF-mCherry*
**Figure 4**	**C–D**	$Nts\text{-}Cre;Ten3^{+/+}$	control	—	—	dSub	*AAV8-CreON-mCherry*
	**E–F, I**	$Nts\text{-}Cre;Ten3^{fl/fl}$	$Ten3^{Sub}$-cKO	—	—	dSub	*AAV8-CreON-mCherry*
**Figure 5**	**A**	*Sim1-Cre;Ai75D/+*	*Sim1-Cre;LSL-ntdT* [1]	—	—	—	—
	**D–E**	$Ten3^{fl/fl};Lphn2^{fl/fl}$	control	—	—	dSub	*AAV8-ChR2-mCherry*
	**F–G**	$Sim1\text{-}Cre;Ten3^{fl/fl};Lphn2^{fl/fl}$	$Ten3^{mMN}$&$Lphn2^{mMN}$-cKO	—	—	dSub	*AAV8-ChR2-mCherry*
	**H–I**	$Sim1\text{-}Cre;Ten3^{+/+};Lphn2^{fl/fl}$	$Lphn2^{mMN}$-cKO	—	—	dSub	*AAV8-ChR2-mCherry*
	**J–K**	$Sim1\text{-}Cre;Ten3^{fl/fl};Lphn2^{+/+}$	$Ten3^{mMN}$-cKO	—	—	dSub	*AAV8-ChR2-mCherry*
**Figure 6**	**C–D**	$Nts\text{-}Cre;Lphn2^{+/+}$	control	—	—	pSub	*AAV8-CreON-GFP*
	**E–F, I**	$Nts\text{-}Cre;Lphn2^{fl/fl}$	$Lphn2^{Sub}$-cKO	—	—	pSub	*AAV8-CreON-GFP*
**Figure 7**	**C–D**	$Ten3^{fl/fl};Lphn2^{fl/fl}$	control	—	—	pSub	*AAV8-ChR2-GFP*
	**E–F, I**	$Sim1\text{-}Cre;Ten3^{fl/fl};Lphn2^{fl/fl}$	$Ten3^{mMN}$&$Lphn2^{mMN}$-cKO	—	—	pSub	*AAV8-ChR2-GFP*
**Figure S1**	**B**	wildtype	—	—	—	dSub<br>pSub	*AAV8-ChR2-mCherry*<br>*AAV8-ChR2-GFP*
	**D**	CD1 wildtype	—	MEC	Biocytin TMR[2]	—	—
	**E**	$Ten3^{HA/HA};Lphn2^{mVenus/mVenus}$	—	MEC	Biocytin TMR[2]	—	—
	**F–G**	$Ten3^{HA/HA};Lphn2^{mVenus/mVenus}$	—	—	—	—	—
**Figure S2**	**A**	CD1 wildtype	control ($Ten3^{+/+}$)	MEC	*LV-CAG-Cre-GFP*	MEC	*AAV8-CreON-GFP*<br>*AAV8-CreOFF-mCherry*
	**B, F, H**	$Ten3^{fl/fl}$	$Ten3^{MEC}$*-cKO*	MEC	*LV-CAG-Cre-GFP*	MEC	*AAV8-CreON-GFP*<br>*AAV8-CreOFF-mCherry*
**Figure S3**	**A–C**	*Nts-Cre;Ai75D/+*	*Nts-Cre;LSL-ntdT*	—	—	—	—
	**D–E**	*Sim1-Cre;Ai75D/+*	*Sim1-Cre;LSL-ntdT*	—	—	—	—
**Figure S4**	**A**	$Ten3^{fl/fl};Lphn2^{fl/fl}$	control	—	—	MEC	*AAV8-CreOFF-mCherry*
	**B**	$Nts\text{-}Cre;Ten3^{fl/fl};Lphn2^{fl/fl}$	$Ten3^{Sub}$&$Lphn2^{Sub}$-cKO	—	—	MEC	*AAV8-CreOFF-mCherry*
**Figure S5**	**A–B**	$Nts\text{-}Cre;Ten3^{+/+}$	control	—	—	dSub	*AAV8-CreON-mCherry*
	**C–D, I**	$Nts\text{-}Cre;Ten3^{fl/fl}$	$Ten3^{Sub}$-cKO	—	—	dSub	*AAV8-CreON-mCherry*
**Figure S6**	**A**	$Ten3^{fl/fl};Lphn2^{fl/fl}$	control	—	—	dSub	*AAV8-ChR2-mCherry*
	**B, F**	$Sim1\text{-}Cre;Ten3^{fl/fl};Lphn2^{fl/fl}$	$Ten3^{mMN}$&$Lphn2^{mMN}$-cKO	—	—	dSub	*AAV8-ChR2-mCherry*
	**C**	$Sim1\text{-}Cre;Ten3^{+/+};Lphn2^{fl/fl}$	$Lphn2^{mMN}$-cKO	—	—	dSub	*AAV8-ChR2-mCherry*
	**D**	$Sim1\text{-}Cre;Ten3^{fl/fl};Lphn2^{+/+}$	$Ten3^{mMN}$-cKO	—	—	dSub	*AAV8-ChR2-mCherry*
**Figure S7**	**B–C**	$Nts\text{-}Cre;Ten3^{+/+};Lphn2^{+/+}$	control	—	—	pSub	*AAV8-CreON-GFP*
	**D–E**	$Nts\text{-}Cre;Ten3^{fl/fl};Lphn2^{+/+}$	$Ten3^{Sub}$-cKO	—	—	pSub	*AAV8-CreON-GFP*
	**J–K**	$Nts\text{-}Cre;Ten3^{+/+};Lphn2^{+/+}$	control	—	—	dSub	*AAV8-CreON-mCh*
	**L–M**	$Nts\text{-}Cre;\ Ten3^{+/+};Lphn2^{fl/fl}$	$Lphn2^{Sub}$-cKO	—	—	dSub	*AAV8-CreON-mCh*

Table S1. Summary of genotypes, viruses, and injections in each experiment, arranged according to figure panel. Related to Figures 1–7 and Figures S1–S7.

[1] ***LSL-***_ntdT_ = *CAG promoter-loxP-stop-loxP-nucleus-targeted tdTomato* in the *Rosa26* locus (a nuclear Cre reporter designated as *Ai75D*)
[2] Biocytin TMR [5-(and-6)-tetramethylrhodamine biocytin] was injected in several time points around birth, rather than exclusively P0
